# The Ebola Virus matrix protein, VP40, requires phosphatidylinositol 4,5-bisphosphate (PI(4,5)P_2_) for extensive oligomerization at the plasma membrane and viral egress

**DOI:** 10.1038/srep19125

**Published:** 2016-01-12

**Authors:** Kristen A. Johnson, Geoffrey J. F. Taghon, Jordan L. Scott, Robert V. Stahelin

**Affiliations:** 1 Department of Chemistry and Biochemistry, the Eck Institute for Global Health, and the Boler-Parseghian Center for Rare and Neglected Diseases, University of Notre Dame, Notre Dame, Indiana, 46556; 2 Department of Biochemistry and Molecular Biology, Indiana University School of Medicine-South Bend, South Bend, Indiana 46617.

## Abstract

VP40 is one of eight proteins encoded by the Ebola Virus (EBOV) and serves as the primary matrix protein, forming virus like particles (VLPs) from mammalian cells without the need for other EBOV proteins. While VP40 is required for viral assembly and budding from host cells during infection, the mechanisms that target VP40 to the plasma membrane are not well understood. Phosphatidylserine is required for VP40 plasma membrane binding, VP40 hexamer formation, and VLP egress, However, PS also becomes exposed on the outer membrane leaflet at sites of VP40 budding, raising the question of how VP40 maintains an interaction with the plasma membrane inner leaflet when PS is flipped to the opposite side. To address this question, cellular and *in vitro* assays were employed to determine if phosphoinositides are important for efficient VP40 localization to the plasma membrane. Cellular studies demonstrated that PI(4,5)P_2_ was an important component of VP40 assembly at the plasma membrane and subsequent virus like particle formation. Additionally, PI(4,5)P_2_ was required for formation of extensive oligomers of VP40, suggesting PS and PI(4,5)P_2_ have different roles in VP40 assembly where PS regulates formation of hexamers from VP40 dimers and PI(4,5)P_2_ stabilizes and/or induces extensive VP40 oligomerization at the plasma membrane.

The Ebola Virus (EBOV) is a lipid enveloped filamentous virus that results in severe hemorrhagic fever in human and non-human primates with up to 90% fatality^1^. EBOV harbors a negative sense RNA genome that only encodes seven genes, one of which is for the viral matrix protein, VP40. VP40 is the most abundant protein in EBOV virions and can form authentic looking virus like particles (VLPs) that bud from the plasma membrane of live cells in the absence of other EBOV proteins^2^,^3^. VP40 is required for the virus to exit from the host cell membrane, suggesting it is a potential drug target^2^,^4^,^5^. In addition to forming the matrix layer in the viral particles, VP40 interacts with several host proteins during trafficking to and while residing at the plasma membrane^6^,^7^,^8^,^9^,^10^,^11^. VP40, which was shown to be a dimer, can also adopt several structural conformations during the viral life cycle including a hexameric filament required for budding^12^,^13^,^14^.

VP40 is a peripheral protein containing 326 amino acids with a N-terminal domain (NTD) that mediates dimerization^12^ and oligomerization^12^,^15^,^16^ and C-terminal domain (CTD) that mediates membrane binding^12^,^17^,^18^. Several studies have shown that VP40 requires both its NTD and CTD, for association with the PM^19^. The association with the plasma membrane anionic interface has been proposed to trigger VP40 structural reorganization into a zigzag hexamer^12^ and subsequently a highly ordered matrix structure that coats the inside of the viral particle^17^,^18^,^19^,^20^,^21^.

VP40 interactions with the plasma membrane are mediated by electrostatic interactions between a CTD basic patch and anionic lipids enriched in the plasma membrane inner leaflet^12^. Additionally, hydrophobic interactions are likely an important part of VP40 plasma membrane assembly, as a hydrophobic loop in the CTD has been found to insert into lipid vesicles^17^,^18^. More than a decade ago, VP40 was shown to associate with phosphatidylserine (PS) containing vesicles^22^,^23^ and more recently it was shown that PS was essential for VP40 plasma membrane localization and formation of VLPs^24^. PS is also required for the formation of hexamers in live cells from VP40 dimers. In addition, PS becomes exposed on the outer leaflet of non-apoptotic cells at sites of VP40 budding. The PS exposure is most abundant on VLPs; this observation was expected as PS exposure expedites entry into host immune cells through interaction with the PS binding TIM-1 receptor^25^,^26^,^27^. This finding, however, raised some questions regarding how VP40 maintains the structural integrity of the viral particle since the VP40 interaction partner PS becomes exposed on the exoplasmic leaflet of VLPs. This lead us to hypothesize that other lipids at the plasma membrane may be involved in coordinating and maintaining the VP40-plasma membrane interactions.

Phosphatidylinositol-4,5-bisphosphate (PI(4,5)P_2_) is this most abundant phosphoinositide (PIP) in mammalian cells comprising up to 2–5% of the plasma membrane^28^,^29^,^30^. The subcellular location of PI(4,5)P_2_ is restricted to the plasma membrane inner leaflet (major)^28^,^31^,^32^ and within the nucleus (minor)^33^,^34^,^35^ of mammalian cells. Several viral matrix proteins including the matrix proteins from human immunodeficiency virus type-1 (HIV-1)^36^, HIV-2^37^, and Mason-Pfizer monkey virus (M-PMV)^38^,^39^ require PI(4,5)P_2_ for association with the PM, matrix assembly, and egress. Each of these matrix proteins utilize a basic patch of residues that coordinate the head group of PI(4,5)P_2_. VP40 also has a highly basic region in its CTD that may interact with PS, PI(4,5)P_2_, or other PIPs at the plasma membrane. To test this possibility, we employed cellular and *in vitro* assays to examine VP40 association with plasma membrane lipids. Results demonstrate that VP40 associates with PI(4,5)P_2_ in the plasma membrane and *in vitro* vesicles, and this interaction is a prerequisite for extensive VP40 oligomerization at the plasma membrane and VLP formation.

## Results

### The VP40-Plasma Membrane interaction is beyond nonspecific electrostatics

**Figure 1:**
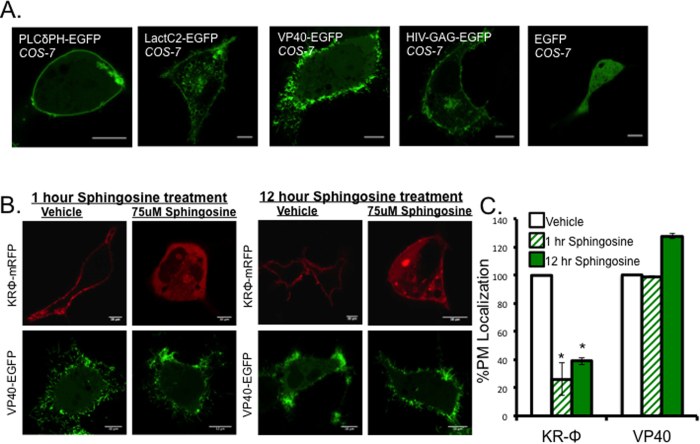
VP40 localizes to the plasma membrane of COS-7 cells. (**A)** Representative images of PLCδPH-EGFP, LactC2-EGFP, EGFP-VP40, and HIV-GAG-EGFP 14 hours post transfection in COS-7 cells. (**B**) Representative images of KRΦ-mRFP and EGFP-VP40 treated with either vehicle (EtOH 1:500) or 75 uM sphingosine for 1 or 14 hours. (**C**) Percent plasma membrane localization of KRΦ-mRFP and EGFP-VP40 following one and 12 hour 75 uM sphingosine treatments. All data was quantified in MATLAB where %PM localization was defined by (PM intensity/total intensity)*100. Data was normalized to the %PM localization of each construct with the 1 or 12 hour vehicle treatment, which was set to 100%. Error bars are ± SEM from at least 20 cells over three independent experiments. All scale bars are 10 μm.

An EGFP fusion tagged version of VP40 localized to the inner leaflet of the plasma membrane of COS-7 cells where VLPs are formed. VLP formation at the plasma membrane was observed as early as 8 hours post transfection in COS-7 cells and were abundant at 14 hours post transfection (Fig. 1A). To determine the average cellular distribution and plasma membrane localization of VP40 and control proteins, PLCδ-PH (PI(4,5)P_2_ biosensor)^40^, Lact C2 (PS biosensor)^41^, and HIV-1 Gag were each transfected into COS-7 cells and imaged 14 hours post transfection with a Zeiss LSM 710 confocal laser scanning microscope (Fig. 1A). Previous work observed no significant changes in VP40 PM localization when the PM was neutralized with sphingosine, a membrane permeant base^41^. To verify VP40 exhibited similar behavior in COS-7 cells, we repeated these experiments using 75 μM sphingosine or an equivalent volume of vehicle (ethanol). Sphingosine or vehicle were added to the media of cells expressing the polybasic charge sensor KRΦ^42^ or VP40 transfected cells for 1 or 12 hours. As previously reported^24^, plasma membrane localization of KRΦ was significantly reduced upon sphingosine treatment, compared to vehicle (Fig. 1B,C) but no significant decrease in VP40 plasma membrane localization was observed with either sphingosine treatment or vehicle control (Fig. 1B,C). These findings indicated that VP40 localized to the PM through a mechanism independent of nonspecific electrostatics in COS-7 cells.

### Competition assays to identify potential VP40-lipid interaction partners

**Figure 2:**
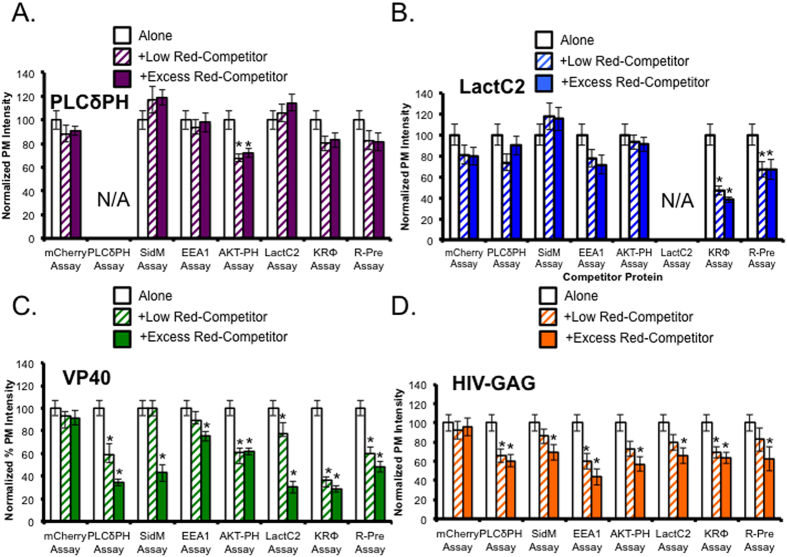
VP40 plasma membrane localization is disturbed by overexpression of various lipid biosensors. **(A**) Percent plasma membrane localization of PLCδ-PH-EGFP construct (0.4 μg) when co-transfected with low levels (0.15 μg) and excess (0.5 μg) competitor protein. (**B**) Percent plasma membrane localization of LactC2-EGFP construct when co-transfected with low levels and excess competitor protein. (**C**) Percent plasma membrane localization of EGFP-VP40 construct when co-transfected with low levels and excess competitor protein. (**D**) Percent plasma membrane localization of HIV-GAG-EGFP construct when co-transfected with low levels and excess competitor protein. Each data set represents the average percent plasma membrane localization of at least 15 independent cells from three independent experiments. All data was quantified in MATLAB where %PM localization is defined by (PM intensity/total intensity)*100. Data was normalized to the %PM localization of each construct when transfected alone in COS-7 cells for the same time (14 hours). Error bars are ± SEM. Significant changes (p < 0.05) from normal plasma membrane localization are marked with a star (*).

To identify potential anionic lipid substrates in addition to PS at the plasma membrane, EGFP-VP40 (0.4 ug DNA) was co-transfected with a low level (0.15 ug DNA) or excess level (0.5 ug DNA) of a lipid biosensor with a mCherry or mRFP tag. This allowed for competition of various lipids including PI(4,5)P_2_ (PLCδ-PH), PI(4)P (SidM-P4M), PI(3)P (EEA1), PI(3,4,5)P_3_ (AKT-PH) and PS (LactC2). Polybasic charge sensors KRΦ (peripheral) and R-Pre (lipidated) were used to mask anionic lipids as a positive control and mCherry alone was used as a negative control. The quantified plasma membrane localization of VP40 and control proteins with low or excess of competitor protein are shown in Fig. 2. VP40 plasma membrane localization was significantly reduced compared to VP40 alone when co-transfected with the PLCδ-PH PI(4,5)P_2_ biosensor, Akt-PH PI(3,4,5)P_3_ biosensor, excess SidM PI(4)P biosensor SidM, excess PI(3)P biosensor EEA1, excess PS biosensor LactC2, and polybasic charge sensors KRΦ and R-Pre. VP40 localization was not significantly decreased when co-transfected with mCherry. Representative images from each assay are shown in supplementary Figure 1. The polybasic charge sensors also produced a significant decrease in VP40 and LactC2 PM localization indicating that the loss in VP40 PM localization is likely due to masking of PS at the PM. Together these findings suggested that VP40 may likely require phosphoinositide (PIP) substrates at the PM in addition to PS. To further test this hypothesis, lipid phosphatase enzymes were co-transfected with VP40 to assess the role of some PIPs in VP40 localization.

### Constitutive depletion of PI(4,5)P_2_ in Live Cells

**Figure 3:**
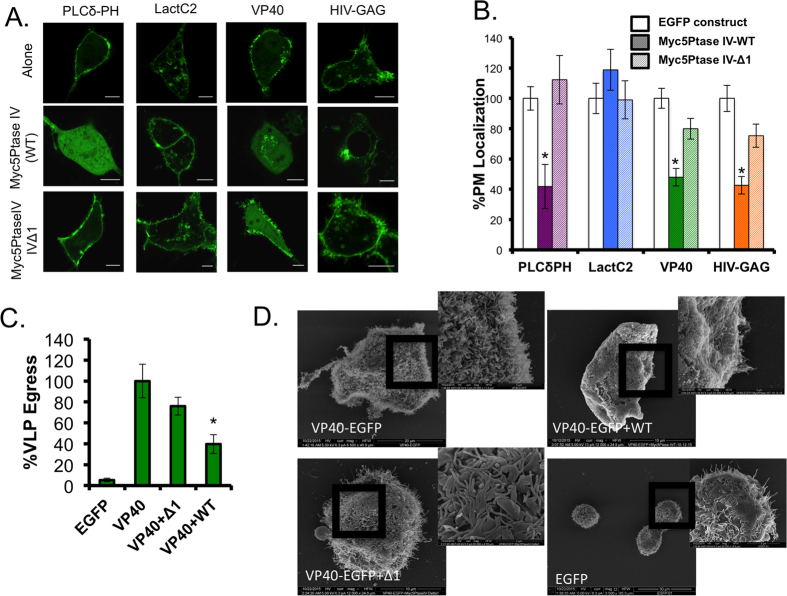
Constitutive enzyme depletion of lipid substrates results in decreased VP40 VLP formation. **(A**) Representative images of EGFP constructs PLCδ-PH-EGFP (N = 20), LactC2-EGFP (N = 20), EGFP-VP40 (N = 24), and HIV-GAG-EGFP (N = 20) co-transfected with Myc-5’PtaseIV-WT, or Myc-5’PtaseIV-Δ1 in COS-7 cells. All scale bars are 10 μm. N indicates the numbers of cells imaged over three independent transfections. (**B**) Quantitative analysis of plasma membrane localization of each construct under each co-transfection condition. All values represent at least 20 independent cells over three independent experiments ± SEM. All data was quantified in MATLAB where %PM localization is defined by (PM intensity/total intensity)*100. Data was normalized to the %PM localization of each construct when transfected alone in COS-7 cells for the same time (14 hours). Significant changes (p < 0.05) from normal PM localization are marked with a star (*). (**C**) A VLP egress assay was performed. VLPs were collected and purified from EGFP-VP40 transfected cells (see methods) and the EGFP content was quantified using a GFP ELISA kit (Cell Biolabs) and the percentage VLP egress was determined as the ratio of GFP in the VLP sample to the total GFP (VLP+cell lysate) sample. Significant changes (p < 0.05) from normal VLP egress are marked with a star. (**D**) Scanning electron microscopy (SEM) of COS-7 cells transfected for 14 hours with VP40, EGFP-VP40 +Myc5’PtaseIV-WT, EGFP-VP40 +Myc-5’PtaseIV-Δ1, or EGFP. Images are shown for each sample, two replicates were performed.

Consitutive enzyme Myc-5’PtaseIV were used to deplete PI(4,5)P_2_. A constitutive 5-phosphatase Myc-5′PtaseIV-WT (active) and Myc-5′PtaseIV-Δ1 (inactive) have been effective tools to investigate the PI(4,5)P_2_ binding properties of various proteins including, HIV-1 Gag^36^. This phosphatase construct is myristoylated post transnationally, which targets it to the plasma membrane. When VP40 was co-transfected with Myc-5′PtaseIV-WT, plasma membrane localization was significantly decreased similar to the response of PLCδ PH in the presence of Myc-5′PtaseIV-WT (Fig. 3A,B). Under similar conditions the HIV-1 GAG exhibited remarkable depletion of PM localization while the PS biosensor Lact C2 did not. In contrast, VP40 PM localization was not significantly reduced in the presence of a the catalytically inactive Myc-5′PtaseIV-Δ1.

To determine if plasma membrane PI(4,5)P_2_ levels were important for VP40 VLP formation, a VLP egress assay was performed 24 hours post transfection when VP40 was co-expressed with either the active or catalytically inactive 5′-PtaseIV. After collecting VLP and cell lysate (CL) samples (see methods section) an ELISA assay (Cell Biolabs) was used to quantify the percent GFP in each sample. A summary of findings are presented in Fig. 3C where VLP egress of EGFP-VP40 was normalized to 100%. This data is in line with the findings from the confocal imaging experiment where PI(4,5)P_2_ was essential for normal VLP formation (Fig. 3C). The residual plasma membrane localization and VLP egress could likely be due to incomplete co-transfection of enzymatic constructs with VP40. To determine if morphological changes existed for VLP formation and to further confirm the importance of PI(4,5)P_2_ in VLP formation, scanning electron microscopy was used to visualize the plasma membrane morphology of cells transfected with EGFP-VP40, EGFP alone, or EGFP-VP40 co-expressed with either Myc5’PtaseIV-WT or Myc5’PtaseIV-Δ1 (Fig. 3D). An abundance of VLPs and smaller bud structures were observed on EGFP-VP40 and VP40 + Myc5’PtaseIV-Δ1 transfected cells. VLPs were not readily observed on VP40 + Myc5’PtaseIV-WT cells, however, smaller bud structures were somewhat visible. EGFP transfected cells did not have significant bud structures but had a visible low abundance of filopodia structures on some cells, which is also observed on some EGFP transfected cells using confocal microscopy (data not shown).

### Pharmacological treatments support VP40 phosphoinositide dependence at the plasma membrane

**Figure 4:**
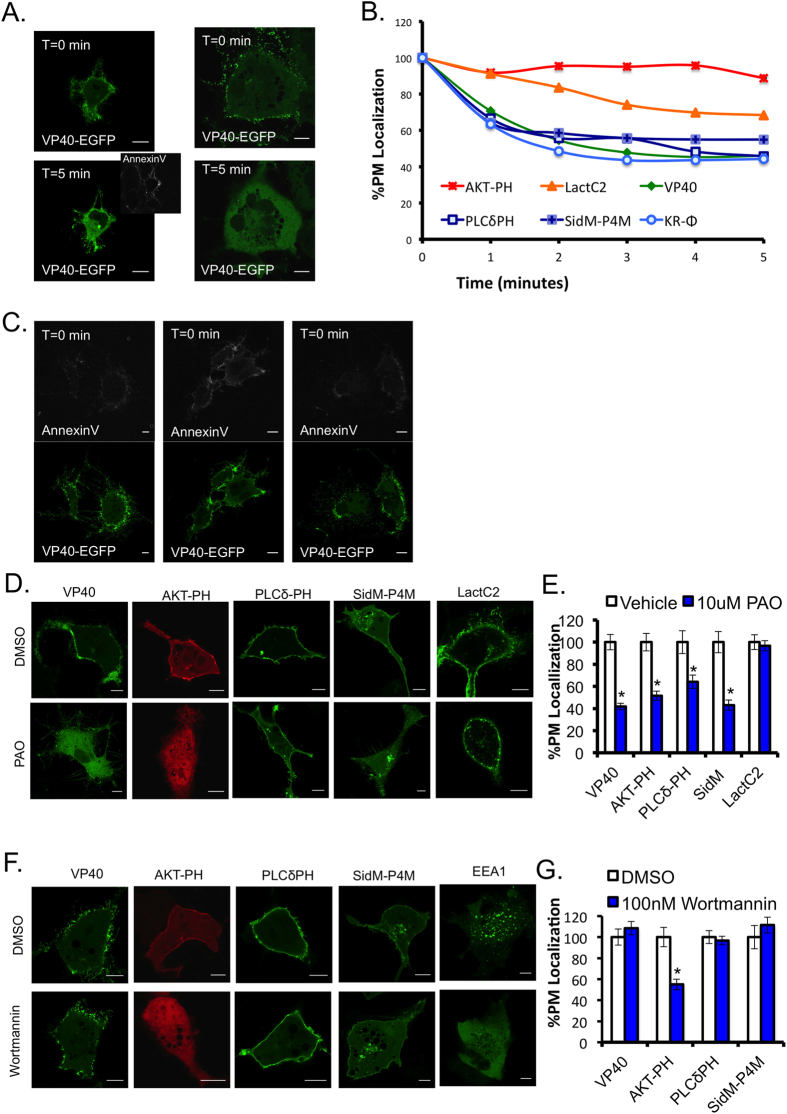
PIP depleting pharmacological treatments yield a decrease in VP40 PM association. (**A**) Representative images of VP40 transfected into COS-7 cells for 14 hours pre-, and 5 minutes post 10 μM ionomycin treatment in 1x Annexin V binding buffer. Annexin V APC was added five minutes post treatment (1:25) and imaged after a five-minute incubation. Scale bars are 10 μm. **(B**) Percent plasma membrane localization of each construct was quantified over a 5 minute 10 μM ionomycin treatment, where data were normalized to pre-treatment plasma membrane localization of 100%. (**C**) Representative images of EGFP-VP40 pre-ionomycin treatment with AnnexinV-APC staining are shown to demonstrate PS exposure in VP40 expressing cells prior to ionomycin addition. (**D**) Representative images of EGFP-VP40 (N = 22), AKT-PH-mRFP (N = 34), LactC2-EGFP (N = 16), PLCδ-PH-EGFP (N = 16) and SidM-P4M-GFP (N = 24) with 30 minute, 10 μM PAO treatment or equal volume DMSO control treatment. Scale bars are 10 μm. (**E**) Percent PM localization of PAO treated cells compared to DMSO treated cells, normalized to 100%. (**F**) Representative images of EGFP-VP40 (N = 20), AKT-PH (N = 18), PLCδ-PH-EGFP (N = 18), SidM-P4M-GFP (N = 22), and EEA1-YFP, with 1 hour, 100 nM Wortmannin treatment or equal volume DMSO control treatment. (**G**) Percent PM localization of Wortmannin treated cells compared to DMSO treated cells, normalized to 100%. Average values ± SEM are shown for each construct, N indicates the numbers of cells imaged over three independent transfections. Significance was determined with a two-tailed T-test, p values less than 0.05 are marked with a star.

To remove various phospholipid substrates without co-transfection, ionomycin, phenyl arsine oxide (PAO) and wortmannin treatments were used. Ionomycin is a calcium ionophore, where increased cellular calcium activates PLC and induces activation of a lipid scramblase that induces some loss of plasma membrane asymmetry. As a result, the PI(4,5)P_2_ and PI(4)P head groups are hydrolyzed by PLC and PS becomes more exposed on the outer surface of the plasma membrane, as a function of scramblase activity^43^. Representative images of VP40 pre-treatment and post-treatment with ionomycin demonstrate VP40 dissociation from the plasma membrane as well as an increase in PS exposure as detected with the extracellular PS biosensor Annexin V (Figs 4A and S2C), The average %PM localization of each construct tested was quantified versus time (Fig. 4B). It was previously reported that VP40 expression in mammalian cells induces PS exposure on the exterior of VLPs in HEK293 cells^24^; this was also observed in EGFP-VP40 transfected COS7 cells before ionomycin treatment (Fig. 4C). Furthermore, VP40 dissociated from the plasma membrane upon ionomycin treatment similarly to PI(4,5)P_2_ and PI(4)P biosensors, PLCδ-PH and SidM-P4M, respectively (Fig. 4B). Images of control biosensors are shown in Figure S2.

PAO, a PI4 kinase inhibitor resulted in a significant decrease in PI(4)P and PI(4,5)P_2_ at the PM as shown with biosensors SidM-P4M and PLCδ-PH (Fig. 4D). Additionally, VP40 PM localization was significantly reduced with PAO treatment versus the DMSO control (Fig. 4D,E). The PI3 kinase inhibitor, Wortmannin, resulted in a significant decrease in AKT-PH plasma membrane localization but had no significant effect on VP40, compared to the DMSO control (Fig. 4F,G). Additionally, the PI(3)P biosensor EEA1-FYVE was displaced from endosomes following Wortmannin treatment but not vehicle control (Fig. 4F). Together, the pharmacological treatments reinforce the hypothesis that phosphoinositides promote VP40 localization at the plasma membrane. However, each of the treatments used have promiscuity in their substrate selectivity and therefore, a more specific lipid depletion method was used to confirm results.

### Real time lipid depletion and lipid binding assays reveal VP40 cellular and *in vitro* phosphoinositide selectivity

**Figure 5:**
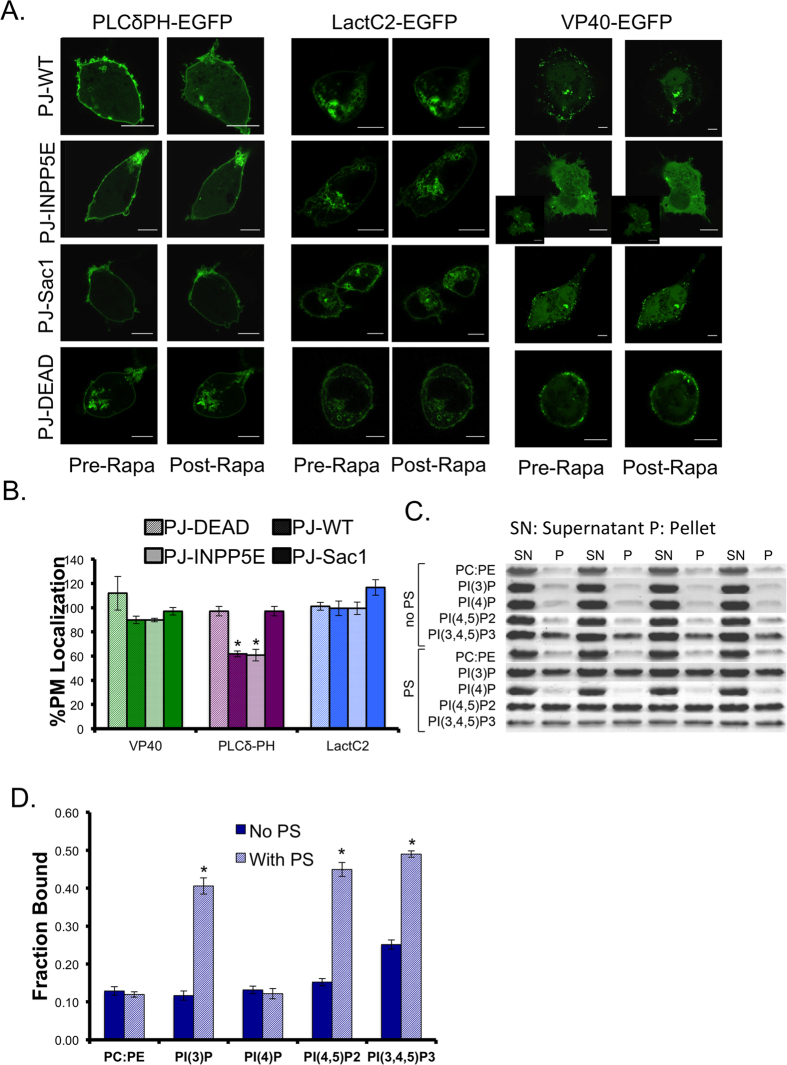
Real time lipid depletion and *in vitro* binding reveal VP40 membrane association is significantly enhanced by PI(4,5)P_2_. (**A**) Representative images of PLCδ-PH-EGFP, LactC2-EGFP, and EGPF-VP40 pre and post rapamycin treatment with PJ-WT-FKBP-mRFP, PJ-INPP5E-FKBP-mRFP, PJ-Sac1-FKBP-mRFP, and PJ-Dead-FKBP-mRFP phosphatase constructs. PM anchor Lyn11-FRB-CFP was used in each experiment. At least 3 independent experiments were used for each condition. (**B**) % PM localization values were plotted for PLCδ-PH-EGFP, LactC2-EGFP, and EGFP-VP40 with depletion by PJ-WT-FKBP-mRFP, PJ-INPP5E-FKBP-mRFP, PJ-Sac1-FKBP-mRFP, and PJ-Dead-FKBP-mRFP phosphatase constructs. % PM Localization was calculated using MATLAB for each cell post rapamycin addition, normalized to pre rapamycin addition. Average values ± SEM are shown for each construct. Significant changes (p < 0.05) for each PJ-construct as compared to those with the PJ-Dead construct are marked with a star. (**C**) Representative SDS PAGE gel images of VP40 in the supernatant (SN) and pellet (P) with each liposome condition, 4 replicates are shown for each condition. **(D**) Average fraction VP40 bound to multilamellar vesicles of various composition with average values plotted ± SEM. Significant changes (p < 0.05) are marked with a star.

A rapamycin inducible lipid depletion system generated by Hammond *et al.* was used to specifically deplete PI(4,5)P_2_ and PI(4)P in real time^44^. VP40 or control proteins (LactC2 or PLCδ-PH) were co-transfected with a plasma membrane anchored Lyn11-FRB-CFP and one of four pseudojanin (PJ)-FKBP cytosolic constructs. Four PJ-constructs were used to deplete PI(4,5)P_2_ (PJ-INPP5E), PI(4)P (PJ-Sac1), PI(4,5)P_2_ and PI(4)P (PJ-WT), or neither (PJ-Dead, inactive)^44^. Representative images of the EGFP construct before and after each experiment are shown in Fig. 5A. The percent plasma membrane localization was calculated before rapamycin addition (set to 100%) and after rapamycin addition, the % PM localization post depletion is shown in Fig. 5B. VP40 had a slight decrease in PM localization when PI(4,5)P_2_ or PI(4)P was depleted, but it wasn’t statistically significant (with PJ-WT, PJ-INPP5E, and PJ-Sac1) compared to the inactive construct (PJ-Dead) (Fig. 5B). The change in VP40 PM localization was much less than the control PLCδ-PH construct. The molecular basis of this finding is unknown, further studies will be required to fully determine the mechanistic differences. Further experiments as described below suggest PI(4,5)P_2_ is a critical determinant of extensive VP40 oligomerization. Additionally, the VLPs present on cells co-transfected with Lyn11 are not as prominent as with non-PM constructs (Fig. 2, Figure S1). When VP40 was co-transfected with Lyn11, PM localization was significantly reduced compared to VP40 expression without Lyn11 (Figure S3A, S3B).

A multilamellar vesicle (MLV) sedimentation assay was used to determine if VP40 binds to PI(4,5)P_2_ and other PIPs *in vitro*. Representative gel images are shown in Fig. 5C and quantified results are shown in Fig. 5D. There is a significant increase in VP40 MLV binding to vesicles containing both PS and either PI(3)P, PI(4,5)P_2_, or PI(3,4,5)P_3_ compared to vesicles harboring PIPs in the absence of PS. Notably, VP40 displayed a ~3-fold increase in binding to PI(4,5)P_2_ containing vesicles when PS was present. VP40 also associated with PI(3)P and PI(3,4,5)P_3_ containing vesicles in this manner but not those containing PI(4)P. While VP40 associates with PI(3)P and PI(3,4,5)P_3_ *in vitro*, PI(3)P is generally found on vesicles during endocytosis^31^ and PI(3,4,5)P_3_ represents only 2–5% of PI(4,5)_2_ levels at the plasma membrane^28^,^45^. Therefore, the combination of cellular and *in vitro* data suggest VP40 association with PI(4,5)P_2_ may be the most important PIP interaction for plasma membrane assembly. Nonetheless, interactions with or the importance of other PIPs cannot be discounted for VP40 as PI(4)P and PI(3,4,5)P3 play some role in PM localization as evidenced by the competition assay and other PIPs (PI(3)P, PI(4)P, or PI(3,5)P_2_) could play some sort of trafficking role in VP40 targeting to the PM.

### PI(4,5)P_2_ is required for VP40 oligomer formation at the plasma membrane

Adu-Gyamfi *et al.* recently demonstrated that PS was required for VP40 oligomerization and VLP formation as PS depleted cells VP40 had a low level of oligomerization compared to the controls^25^. Moreover, the inhibition of VP40 oligomerization was quite apparent as Number and Brightness analysis (N&B) revealed PS was essential for significant oligomerization to occur beyond the constitutive VP40 dimer. To determine the role of PI(4,5)P_2_ in VP40 oligomerization, raster image correlation spectroscopy (RICS) and N&B analysis were used to investigate the diffusion coefficient and average oligomer size of VP40 at the plasma membrane with or without PI(4,5)P_2_ depletion.

**Figure 6:**
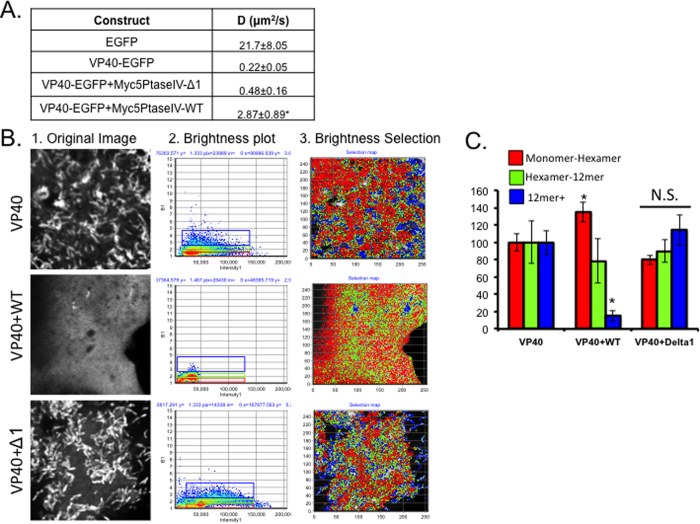
VP40 diffusion and oligomerization near the plasma membrane of the COS-7 cells were modified after PIP depletion. **(A**) Diffusion coefficients for EGFP (N = 4), EGFP-VP40 (N = 9), EGFP-VP40+Myc-5’PtaseIV-Δ1 (N = 4) and EGFP-VP40+Myc-5′PtaseIV-WT (N = 8) were calculated using raster image correlation spectroscopy (RICS) in SimFCS. (**B**) Representative images from EGFP-VP40 (N = 14), EGFP-VP40 + Myc-5 ‘PtaseIV-WT (N = 11), and EGFP-VP40 +Myc-5′PtaseIV- Δ1 (N = 11) number and brightness workflow in SimFCS. The original intensity image is shown in panel 1, the number verses intensity plot in panel 2 and the selection plot of the cell image in panel 3. (**C**) The percent pixels with a brightness value of 1–1.5 (monomer-hexamer), 1.5–2.0 (hexamer to 12mer) and 2.0 ~ 6.5 (12mer to ~36mer) were calculated per cell and the average value for construct was plotted. Average values shown for all graphs ±SEM. Significant changes (p < 0.05) are marked with a star.

The diffusion coefficient of EGFP-VP40 was determined for each sample at a region near the surface of each cell using an Olympus FV1000 2-photon confocal microscope. For each cell, a stack of 100 images was collected according to specifications in the methods section. Data analysis in SimFCS was then performed to determine the diffusion coefficient and the oligomerization state of EGFP-VP40 or EGFP. The diffusion coefficient of EGFP-VP40 and EGFP-VP40 co-transfected with the catalytically inactive Myc-5’PtaseIV construct had no significant differences (Fig. 6A). However, when VP40 was co-transfected with the WT phosphatase Myc-5’PtaseIV, the diffusion coefficient of EGFP-VP40 was significantly increased compared to the diffusion coefficient of EGFP-VP40 with the inactive phosphatase (Fig. 6A).

Number and Brightness analysis (N&B) is a method that measures the variance in intensity at each pixel over time (100 image stack) to determine the intensity and brightness of the fluorescent protein at each pixel throughout the image^46^. As a result, the intensity at each pixel is distinguished from the oligomerization state at each pixel and the results are shown in a brightness (B) vs. intensity plot as shown in Fig. 6B, panel 2. EGFP was used to determine the true brightness of a monomer in COS-7 cells; a multiple of this value indicates an increase in protein oligomerization. The pixels correlating to monomer-hexamer, hexamer-12mer, and 12mer and larger were calculated and average (see Equation 1). An image representation of this is shown in Fig. 6B, panel 3. The percentage of pixels in each bin were averaged and plotted for EGFP-VP40, EGFP-VP40 + Myc5’PtaseIV-WT, and EGFP-VP40 + Myc5’PtaseIV-Δ1 (Fig. 6C). VP40 co-transfected with WT Myc-5’PtaseIV exhibited a significant decrease in the percentage of 12mer or greater near the surface of the cells and an increase in monomer-hexamer, compared to EGFP-VP40 alone. There was no significant difference in oligomerization between EGFP-VP40 and EGFP-VP40 + Myc5’PtaseIV-Δ1 (Fig. 6C). These data indicate that PI(4,5)P_2_ is an important component of stabilization of VP40 filaments at the plasma membrane surface at, which is likely a critical step in forming the extensive VP40 matrix layer that completely underlies the viral lipid envelope.

## Discussion

VP40 is the most abundant protein in EBOV virions and is required for viral particle formation and egress. Because of its critical role in the life cycle of the virus, VP40 has been suggested as a potential therapeutic target^4^,^47^. More than a decade ago, VP40 was shown to associate with lipid vesicles containing PS^22^,^23^ as well as lipid raft regions of the plasma membrane^48^. Additionally, VP40 is able to associate with PS containing vesicles in the absence of phosphoinositides, a necessary step in promoting VP40 oligomerization of VP40 dimers^22^,^23^,^24^. However, until recently, mechanistic information of VP40 interactions with lipids was limited. First, VP40 was shown to interact with the hydrocarbon core of lipid membranes containing PS, and this interaction was shown to be important for VLP formation^17^,^18^. Recently, cellular studies by Adu-Gyamfi *et al.* demonstrated that cellular PS is essential for VP40 plasma membrane localization, oligomerization, and VLP egress^24^. However, it was unclear whether VP40 required additional lipid substrates to maintain the structural integrity of the virus particle when PS was exposed to the extracellular side of the VLP.

Several viral matrix proteins selectively bind PI(4,5)P_2_ at the PM^36^,^37^,^49^. This phosphoinositide is enriched at the PM, making up over 99% of the bis-phosphorylated species^29^,^50^. While VP40 can associate with PS containing membranes lacking PI(4,5)P_2_, we have shown that this lipid is required for normal VP40 localization to the plasma membrane and large oligomer formation in COS-7 cells (Fig. 2C, Supplementary Figure 1, Figure 3, Figure 4, Figure 5). The loss of plasma membrane localization was not simply due to a decrease in the overall membrane anionic charge; VP40 plasma membrane localization was not decreased when the plasma membrane was neutralized (Fig. 1B,C). When KR-Φ or R-Pre were over expressed with VP40, there was significantly less VP40 at the PM, likely because PS was masked by the polybasic probes as LactC2 PM localization was also reduced (Fig. 2B,C, Supplemental Figure 1).

MLV binding analysis indicated that VP40 associates with PI(4,5)P_2_ as well as PI(3)P and PIP_3_ (Fig. 5C,D). These interactions were weak in the absence of PS in the MLVs but significantly increased when PS was present. In contrast, binding to PI(4)P was not evident indicating VP40 may harbor some PIP binding selectivity. While VP40 binds PI(3)P containing vesicles *in vitro*, PI(3)P may not have a direct role in VP40 plasma membrane localization (Fig. 4F,G). From the combination of cellular and *in vitro* work, the current data suggests that PI(4,5)P_2_ is important for VP40 plasma membrane localization, extensive oligomerization and stabilization of VP40 oligomers at the plasma membrane surface, as well as VLP egress. Interestingly, PS was previously shown to be critical for forming oligomers larger than the dimer^24^ and here we find that PI(4,5)P_2_ was critical for oligomers 12mer and greater but not likely those between a dimer and 12mer. While additional investigation is required to fully resolve how PS and PI(4,5)P_2_ structurally act as molecular switches in VP40 oligomerization, it seems these lipids have distinct roles in VP40 assembly. We propose PS is essential for the initial association of VP40 with the plasma membrane and subsequent oligomerization of the dimer. As PS exposure increases on the outer leaflet of the plasma membrane, we propose PI(4,5)P_2_ is important for stabilizing the extensive VP40 oligomers that form and may be a key regulator of VP40 concatenation to form the underlying protein matrix layer.

VP40 inserts a CTD hydrophobic loop into the plasma membrane upon association^17^,^18^; this increases residence time of lipid binding and is likely an essential step in structural rearrangement of VP40 oligomers. It is unknown if this event is induced or strengthened by a specific lipid substrate or if PS binding to VP40 is a prerequisite for PI(4,5)P_2_ binding and oligomerization. HIV-1 Gag proteins harbor a myristate required for membrane retention^51^ and also associate with PI(4,5)P_2_, which is required for assembly and budding^36^,^52^,^53^. Further, Gag multimerization can increase membrane affinity^54^ while membrane association of Murine Leukemia Virus Gag is greatly enhanced with increasing PS^49^. Although VP40 is not known to have a lipidation site, the CTD hydrophobic loop insertion^17^,^18^ could require a specific lipid trigger for exposure and membrane association. This mechanism of insertion may also help to explain the increased membrane retention of VP40 compared to PLCδ-PH in the rapamycin induced PIP depletion experiments. PLCδ-PH interactions with the plasma membrane surface are primarily through interactions with the anionic surface containing PI(4,5)P_2_. We hypothesize that in the absence of PI(4,5)P_2_ VP40 would still be able to interact with PS, harbor hydrophobic interactions with the lipid acyl chains, and may have higher avidity due to its oligomerization (hexamer to 12mer).

The differential roles of PS and PI(4,5)P_2_ in localization to the plasma membrane and oligomerization have yet to be fully elucidated during VP40 trafficking, plasma membrane association and egress. In 2013, Bornholdt *et al.* identified a cationic patch on the CTD enriched in lysine residues that was required for plasma membrane association^12^. Additional investigation of the residues within the cationic patch may clarify which residues are important for PS, PI(4,5)P_2_, or association with other PIPs. Several retroviruses also interact with PS and PI(4,5)P_2_, but the PS binding site can be blocked by RNA association^55^,^56^, which has been proposed to allow proper and specific targeting to the plasma membrane as PS is found on the cytoplasmic face of endomembranes^57^,^58^. If such a mechanism exists for VP40 due to the potential of its basic patch to associate with RNA is still unknown.

VP40 interactions with other PIPs in the life cycle of the virus cannot be discounted. In our *in vitro* assays we observed VP40 to associate with PI(3)P and PI(3,4,5)P_3_ when PS was present, in a similar fashion to that of PI(4,5)P_2_. Additionally, competition assays demonstrate blocking access to PI(4)P and PI(3,4,5)P_3_ also lowered VP40 plasma membrane localization. PI(4,5)P_2_ levels were clearly important for plasma membrane localization of VP40 as well as VLP egress, which is likely attributed to the role of PI(4,5)P_2_ in stabilizing the extensive VP40 oligomers that form at the plasma membrane. Nonetheless, PI(4)P and PI(3,4,5)P_3_ at the plasma membrane could also play a role in stabilization of VP40 oligomers and VP40 membrane retention time.

EBOV or VP40 could also alter cellular lipid metabolism in its favor by regulating a lipid kinase (e.g., PI3K) or inhibition of a lipid phosphatase (e.g., PTEN). Increases in respective PIPs could then promote binding of distinct structural forms of VP40. For instance, VP40 forms an octameric ring structure that regulates viral transcription^12^ and lipid binding of the ring structure has not been explored. PIP interactions with the octameric ring could also be an important part of the viral life cycle as VP40 has been shown to enter the nucleus^59^ where several PIPs reside. Finally, lipids may also play a role in VP40 trafficking to the plasma membrane^60^. Currently, the role of PS or other PIP species in intracellular VP40 transport is unknown. In the experiments presented here and previously^24^ a decrease in available PS yields a decrease in VLP egress and localization (Fig. 2); however, PS localization is not limited to the plasma membrane. Therefore, PS on secretory vesicles from the Golgi or endocytic recycling vesicles could also have an important role in VP40 trafficking to the plasma membrane. Additionally, while VP40 does not bind to MLVs containing PI4P (Fig. 5C,D), excess SidM-P4M blocks VP40 PM localization. It is still possible that PI(4)P has an indirect role in VP40 trafficking. In contrast, VP40 does bind the monophosphate PI(3)P (Fig. 5C,D), which could have an important role in the life cycle of the virus or VP40 function, independent of plasma membrane assembly and budding as PI(3)P is abundant on early endosomes^28^ and important for autophagy.

There is still much to learn in how EBOV and VP40 interacts with host cell lipids and lipid synthesizing machinery. A greater understanding of these interactions should provide greater insight into the function of lipids in VP40 trafficking, plasma membrane localization, oligomerization, viral particle assembly, and viral egress. Resolving the molecular basis of lipid head group and membrane bilayer association of VP40 with high-resolution structural techniques could provide a more complete picture of the mechanisms by which VP40 structures assemble and rearrange at the plasma membrane inner leaflet.

## Materials and Methods

### Plasmids

The VP40-pET46 expression vector was a kind gift from Erica Ollmann Saphire (The Scripps Research Institute) and EGFP-VP40 was prepared as previously described^21^. The Lact-C2 EGFP, Lact-C2 mCherry, KRΦ-mRFP, and Rpre-mRFP plasmids were kind gifts from Dr. Sergio Grinstein (University of Toronto). PLC□ PH GFP was a gift from Tamas Balla (NIH) while the lyn11-FRB-CFP, PJ-WT-FKBP, PJ-INPP5E-FKBP, PJ-Sac1-FKBP, and PJ-Dead-FKBP and SidM-P4m-GFP, SidM-P4Mx2-GFP, SidM-P4M-mCherry were kind gifts from Tamas Balla (NIH) and Gerald Hammond (University Of Pittsburgh). Akt1 PH GFP was from Tobias Meyer (Stanford University), HIV-1 GAG EGFP was a kind gift from Tom Hope (Northwestern University). All plasmids were transformed into DH5α cells and purified using an MIDI-Prep Kit (QIAGEN (Valencia, CA)) with endotoxin free buffers (QIAGEN).

### Cell Culture and Transfection

COS-7 cells were used for all experiments. They were maintained at 37 °C in a 5% CO_2_ humidified incubator supplemented with DMEM with 1 g/L L-Glutamine, D-Glucose, 110 mg/mL Sodium Pyruvate (Life Technologies (Carlsbad, CA)) containing 10% FBS (Sigma (St. Louis, MO)) and 1% Penicillin-Streptomycin (Life Technologies). In preparation for imaging experiments cells were washed with 10 mL 1X PBS, pH 7.4 and trypsinized with 1X Trypsin-EDTA (MP Biomedicals (Santa Ana, CA)). Cells were then seeded in No. 1.5 glass bottom 8 well plates (MatTek (Ashland, MA) and grown to 80–90% confluency before transfection. Cells were transfected with endotoxin free plasmid DNA using Lipofectamine 2000 (Life Technologies) in optiMEM (Life Technologies) according to manufacturers protocol for 14 hours at 37 °C unless indicated otherwise. 0.4ug of DNA was used of each plasmid per transfection for pharmaceutical or lipid treatment. In experiments with one or more plasmid per cell 0.4ug DNA was used for the construct being tested, 0.15 ug DNA in “low” transfection for the competition assay and 0.5 ug for the “excess” transfection in the competition assay. 0.5 ug DNA were used for the enzymatic constructs used in combination with VP40 and other proteins (0.4 ug DNA). For VLP collection experiments and SEM experiments the amount of DNA and Lipofectamine 2000 were scaled up proportionally with sample size.

### Confocal Microscopy

Imaging experiments were performed with a Zeiss LSM 710 inverted microscope using a Plan Apochromat 63× 1.4 numerical aperture oil objective. A 458 nm argon laser was used to excite CFP, 488 nm argon laser was used to excite GFP/EGFP and a 561 nm diode laser was used to excite RFP and mCherry. Laser power was kept constant for all experiments. Three independent experiments were performed for all experiments unless indicated otherwise.

### Image Analysis

ImageJ or MATLAB (MathWorks (Natick, MA)) were used for all data analysis. A custom MATLAB script (available upon request) was used to determine the average intensity at the PM and cytosol of each cell. In the script the average intensity in the plasma membrane (defined by the outer 6 pixels of each cell) and the cytosol (region within the PM region) were calculated. The values for PM and cytosol intensity for each cell were then used to calculate the %PM localization. The %PM localization was calculated with the following equation: PM intensity over the total (PM+Cytosol) intensity ×100%. Values were normalized to controls (control set to 100%) and the standard error of the mean was scaled accordingly. Because this script uses the outer 6 pixels of each cell, a value of 0% PM localization cannot be achieved. When cytosolic EGFP is transfected into COS7 cells, there is a value of 14.8 ± 1.2% PM localization, N = 10. For EGFP-VP40, the %PM localization before normalizing to 100% was 36.3 ± 2.3%, N = 22. When compared to cytosolic EGFP, if EGFP-VP40 has a %PM localization of 14.8 ± 1.2%, there is no true localization. When the data is normalized to 100%, EGFP-VP40 value of 40% PM localization or lower is equivalent to cytosolic EGFP when EGFP is normalized to VP40 (14.8/36.3 = 40.7). A value below 40% (normalized) is usually observed in cells with high intensity structures in the cytosol or internal membranes of the cell. The standard error of the mean was calculated for all datasets and scaled proportionally to the normalized data (SEM*(Normalized Value/Original Value) = Scaled SEM). Data sets are shown ± scaled SEM. A two tailed students t-test was used to determine significance (p < 0.05).

### VLP Collection

COS-7 cells were grown to 90 percent confluency in 100 mm plates and transfected with Lipofectamine 2000 (Life Technologies) and endotoxin free plasmid DNA in optiMEM (Life Technologies) for 24 hours. Media from the transfected plates were removed to harvest VLPs plates were then washed with 10 mL 1x PBS, pH 7.4. VLP Samples (media + 1×PBS wash) were centrifuged for 6 minutes at 4 °C at 900 rpm (Eppendorf 5810 R 15 Amp version) to remove dead cell debris from the sample. Supernatant was then gently pipetted on top of a 20% sucrose cushion and samples were then centrifuged for 120 minutes at 100,000 × *g*, 4 °C (Beckman Coulter Avanti J-30I centrifuge) to pellet VLPs. VLPs were dried and resuspended in 150 mM ammonia bicarbonate (NH_4_HCO_3_) and stored at −80° C. During the 120 minute VLP centrifugation step, cells were trypsinized off of the plates and pelleted for 6 minutes at 4 °C at 900 rpm. Pellets were washed with 1x PBS and cells were counted (BioRad TC10 Automated Cell Counter). Cells were then pelleted again and supernatant was removed. Pellets were then resuspended in a COS-7 specific lysis buffer adapted from Thermo Scientific (50 mM TRIS-HCl, 150 mM NaCl, 1 mM EDTA, 1% TritonX, 0.1% SDS, 2 uM PMSF, 1% Deoxycholic acid, and 1% 100 × protease inhibitor cocktail). Cells were incubated on ice in the lysis buffer for 60 minutes with vortexing every 15 minutes. Cell lysate (CL) samples were centrifuged at 25,000 × *g* for 17 minutes at 4 °C (Beckman Coulter Avanti J-30I centrifuge). The soluble fraction was removed and stored at −80 °C.

### GFP ELISA

A GFP ELISA assay (Cell Biolabs (San Diego, CA)) was used to quantify the GFP in the VLP and cell lysate (CL) samples from VLP collections. The GFP ELISA was performed according to manufactures instructions. ELISA plates were analyzed by measuring absorbance of each microwell on a spectrophotometer (Molecular Devices SpectraMax M5) using 450 nm as the primary wave length. Protein content of VLP and CL samples were determined using a BCA assay (Pierce (Grand Island, NY)) to assist in sample VLP and CL sample dilution. All samples were diluted in assay diluent between 40 and 5000 times to get sample between 10–2000 pg GFP (linear range of this GFP ELISA kit). All standards and samples were performed in duplicate. The %VLP egress was quantified by determining the GFP concentration in the VLP sample over the total GFP (VLP+CL) *100% for each sample. Data represents at least three independent transfections with VLP collections on at least two different days.

### Scanning Electron Microscopy (SEM)

Samples were prepared for SEM by transfecting COS-7 cells using the transfection procedure described in cell culture and transfection. 14 hours post transfection cells were scraped from the tissue culture dish, pelleted and washed with 1× PBS. After pelleting again, cells were resuspended in primary fixative (2% glutaraldehyde, 2% paraformaldehyde in 0.1 M cacodylate buffer pH 7.35) and incubated for at least 14 hours at 4 °C on an orbital rocker. Samples were then pelleted and resuspended in 0.1 M cacodylate buffer and samples were applied to poly-L-lysine coated 13 mm coverslips. 1% osmium tetroxide was applied to samples, followed by water washes, and dehydration using ethanol. Samples were they dried using a Tousimis Autosamdri-931 (Rockville, MD). Before imaging, 3 nm iridium was applied to the samples, images were collected on a Field Emission Scanning Electron Microscope Magellan 400 (FEI) (Hillsboro, OR) with assistance from Tatyana Orlova and the Notre Dame Integrated Imaging Facility.

### Pharmacological and Lipid Treatments

Plasma membrane neutralization: D-erythro-Sphingosine (Avanti Polar Lipids Inc.) was dried under N_2_ and resuspended in ethanol. Cells were treated with 75 uM sphingosine or ethanol vehicle (1:250 vol/vol) for 1 or 12 hours at 37 °C. Data were normalized to ethanol vehicle. Ionomycin treatment: cells were treated with 10 uM ionomycin (Calbiochem (San Diego, CA)) in DMSO or DMSO vehicle were added to cells in glucose free media with 2 mM CaCl_2_ or 1× Annexin V binding buffer (BD Biosciences (Franklin Lakes, NJ)) for five minutes. Cells were imaged pre-treatment and each minute post treatment for a timely quantitative analysis. Annexin V-APC (BD Biosciences) 1:40 was added five minutes post treatment and imaged to confirm PS exposure. Phenylarsine oxide (PAO): Cells were treated with 10 uM PAO (Sigma) in DMSO or DMSO vehicle were added to transfected cells for 30 minutes. Data was normalized to vehicle treatment. Cells were treated with 100 nM Wortmannin (Sigma) or equivalent volume DMSO for one hour at 37 °C.

### Rapamycin Induced Lipid Depletion System

Transfected cells with Lyn11-FRB-CFP, PJ-(WT, INPP5E, Sac1 or Dead)-FKBP-mRFP, and either PLCδ-PH-EGFP, LactC2-EGFP, EGFP-VP40 or HIV-GAG-EGFP were treated with 1 uM (final concentration) rapamycin (Sigma) for 7 minutes during imaging on a Zeiss LSM 710 as described above. Data analysis was performed in MATLAB as described above. The %PM localization was determine pre and post rapamycin addition.

### Protein Purification

Ebola WT-VP40 in the pET46 Ek/LIC (His-tagged) (Novagen) vector was expressed and purified as previously described by Bornholdt *et al.*^12^. Bacterial pellets were lysed with a combination of lysozyme and sonication. The protein was purified by binding with Ni/NTA resin slurry and centrifugation at 400 × g. Purified protein was dialyzed into (1.5 M KCl, 250 mM HEPES, pH 7.4) buffer and concentrated with a 10,000 MWCO filter. A sample of the pure protein was run on an SDS-PAGE gel and Coomassie stained to confirm appearance of a strong band at 40 kDa. Concentration was calculated with a BCA Assay and the concentrated protein stored at 4 °C for a maximum of 14 days.

### Multilamellar Vesicle (MLV) Sedimentation Assay

MLV emulsion was prepared by combining phosphatidylcholine (POPC), phosphatidylethanolamine (POPE), phosphatidylserine (POPS), and PIPs in the specified proportions and drying to a film under nitrogen. The film was then hydrated with VP40 dialysis buffer (150 mM KCl, 25 mM HEPES, 0.1 mM EGTA, pH 7.4) to a lipid concentration of 1.0 mM and incubated for 10 minutes at 37 °C to expedite lipid hydration. The hydrated lipids were vortexed for 60 seconds to form a suspension of MLVs. Suspension and protein were combined to a final protein concentration of 5uM and final volume of 100uL per sample. The mixture was incubated at room temperature for 20 minutes. Following centrifugation at 75,600 × *g* for 20 minutes, the supernatant (soluble protein) was collected. The pellet (lipid associated protein) was resuspended in VP40 dialysis buffer to the same volume as the supernatant and vortexed for 15 seconds. 40 uL of each sample was loaded onto an SDS-PAGE gel and run for 60 minutes at 150 V, 400 mA. Gels were stained with Coomassie Brilliant Blue, imaged and analyzed with ImageJ software to determine percentage of lipid associated protein. The experiment was performed with four replicates per MLV composition and experimental triplicate (n = 12).

### Raster Image Correlation Spectroscopy (RICS)

Images acquired for RICS analysis were collected on an Olympus FluoView FV1000 microscope with a 40 × 1.3 numerical aperture oil objective. Raster scanning with 12.5 μs pixel dwell, and 16.4 zoom (for 50 nm pixel size) was used to collect 100 images of each cell according to the methods described by Rossow *et al.*^61^. Data analysis was performed in SimFCS software according to the protocol of Rossow *et al.*^61^. GFP in solution was used to calibrate the focal volume of the laser beam waist (W_o_) each day data was collected. The diffusion of GFP in solution is known^61^ and held constant to determine the W_o_ value between 0.2 and 0.4. Once W_o_ is determined, it is held constant and diffusion coefficients are determined for each construct. The diffusion coefficients are averaged over three independent experiments and the standard error of the mean was calculated for each and significance determined using a two-tailed t-test.

### Number and Brightness Analysis

Images acquired for N&B analysis were collected as described for RICS. Data Analysis was performed in SimFCS software using the number and brightness analysis tool. This method allows for the discrimination between intensity of a pixel and the brightness of a pixel by measuring the variance of intensity at each pixel over time. This method determines the oligomerization status (brightness) versus intensity of each pixel within the image that can be used to determine various oligomer states of a protein throughout a cell^46^. True brightness of a monomer in COS7 cells was determined using monomeric EGFP (N = 11). A value of 0.122 ± 0.017 was calculated from the peak of the Gaussian brightness distribution plot in SimFCS when the S factor was set to 2.5. Equation 1 was used to calculate aggregate^62^.


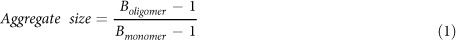


The number of pixels of monomer-hexamer, hexamer-12mer, and 12mer+ were calculated for each condition and normalized to the control, EGFP-VP40. The standard error of the mean (SEM) was calculated for each and significance was determined using a two-tailed t-test. Average values are shown ± SEM.

## Additional Information

**How to cite this article**: Johnson, K. A. *et al.* The Ebola Virus matrix protein, VP40, requires phosphatidylinositol 4,5-bisphosphate (PI(4,5)P_2_) for extensive oligomerization at the plasma membrane and viral egress. *Sci. Rep.* **6**, 19125; doi: 10.1038/srep19125 (2016).

## Supplementary Material

Supplementary Information
