# Supplementary material for: The Ebola Virus matrix protein, VP40, requires phosphatidylinositol 4,5-bisphosphate (PI(4,5)P2) for extensive oligomerization at the plasma membrane and viral egress
Source: Sci Rep. 2016 Jan 12;6:19125. doi: 10.1038/srep19125 (PMC4709572; doi:10.1038/srep19125)
Supplement: Supplementary Information [file srep19125-s1.doc]

**Supplemental Material**

**Title:**

The Ebola Virus matrix protein, VP40, requires phosphatidylinositol 4,5-bisphosphate (PI(4,5)P_2_) for extensive oligomerization at the plasma membrane and viral egress

**Authors:**

Kristen A. Johnson^a^ , Geoffrey J. F. Taghon^b^, Jordan L. Scott^a^, and Robert V. Stahelin^a,b,*^

**Figure S1: Representative Images of VP40 and control protein plasma membrane localization with low expression and overexpression of various lipid biosensors. A.** Representative images of EGFP tagged PLCδ-PH, LactC2, VP40, and HIV-Gag co transfected with low levels of mCherry or mRFP tagged competitor proteins PLCδ-PH, LactC2, SidM-P4M, KR-ϕ, R-Pre, mCherry, or EEA1 for 14 hours, scale bars are 10μm. **B.** Representative images of EGFP tagged PLCδ-PH, LactC2, VP40, and HIV-Gag co transfected with excess levels of mCherry or mRFP tagged competitor proteins PLCδ-PH, LactC2, SidM-P4M, KR-ϕ, R-Pre, mCherry, or EEA1 for 14 hours, scale bars are 10μm. At least three independent transfections were performed for each, N>18 for all.

Figure S2: **Representative Images of additional controls in imaging experiments. A.** Representative images of PLCδ-PH, LactC2, KR-Φ, SidM-P4M, AKT-PH, and EGFP transfected cells treated with Ionomycin pre treatment, 2 minutes post treatment and 5 minutes post treatment. AnnexinV-APC was added 5 minutes post treatment to detect PS exposure in PLCδ-PH and LactC2 transfected cells. All scale bars are 10μm.

Figure S3: **VP40 PM competition with Lyn11:** **A**. Representative images of VP40-EGFP (0.4 ug) and Lyn11-mCherry (0.15ug (Low) or 0.5 ug (Excess)), scale bars are 10 um. **B.** Normalized PM Localzation of VP40 with no Lyn11 (N=21), low Lyn11 (N=16) and excess Lyn11 (N=17). Data shown ± SEM with significance shown with a star (*) determined with a two tailed students t test, P<0.05.

Figure S4: **Number and Brightness Analysis of EGFP: A.** EGFP transfected cell (panel 1) brightness plot (panel 2) and brightness selection (panel 3). 11 EGFP transfected cells were used to calibrate true brightness (0.122) with an S Factor of 2.5 in SimFCS.

**Figure S1.**
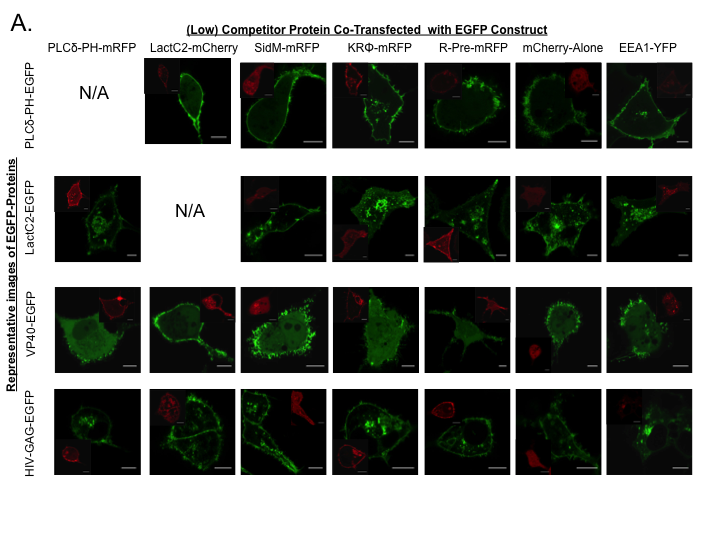

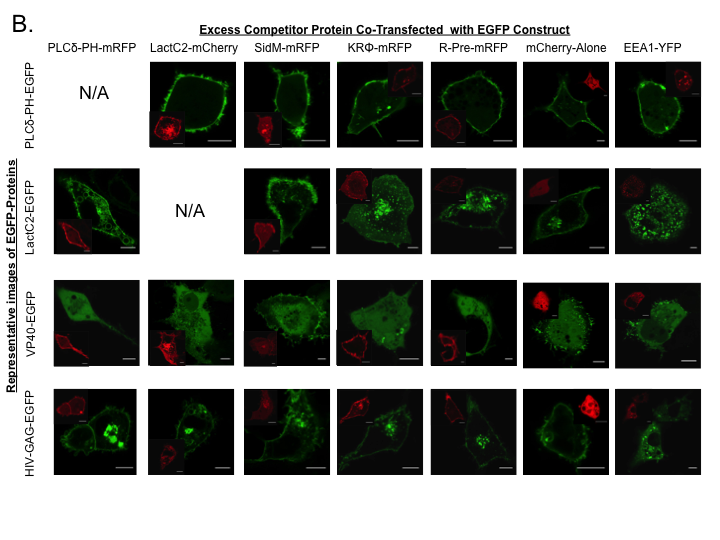


**Figure S2.**


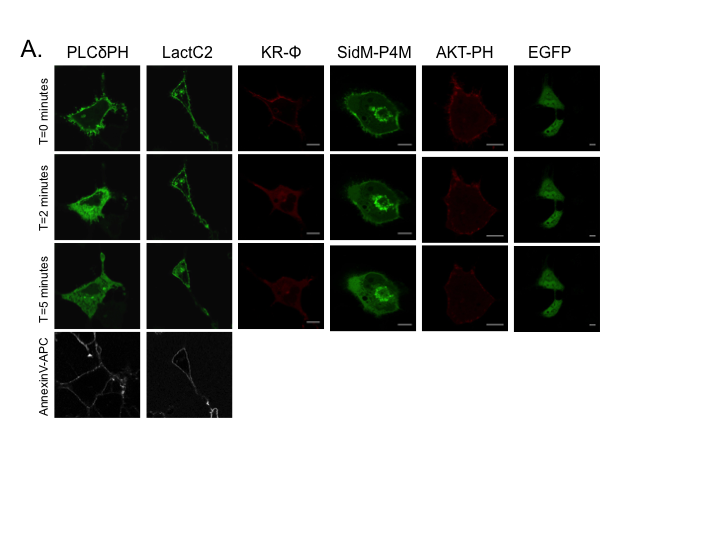


**Figure S3.**


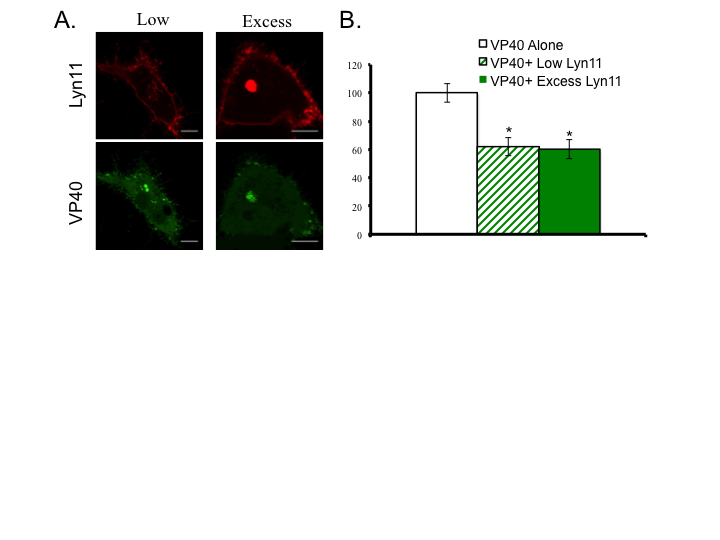


**Figure S4.**

**
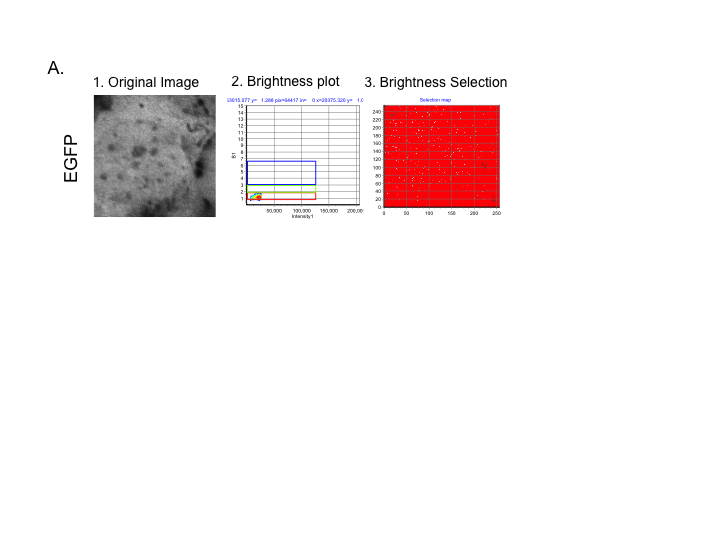
**
